# Another potential zoonotic threat? Herpes B virus in the spotlight

**DOI:** 10.1016/j.nmni.2024.101422

**Published:** 2024-05-07

**Authors:** Francesco Branda, Alessandra Ciccozzi, Marta Giovanetti, Chiara Romano, Massimo Ciccozzi, Fabio Scarpa

**Affiliations:** Unit of Medical Statistics and Molecular Epidemiology, Università Campus Bio-Medico di Roma, Rome, Italy; Department of Biomedical Sciences, University of Sassari, Sassari, Italy; Department of Sciences and Technologies for Sustainable Development and One Health, Università Campus Bio-Medico di Roma, Italy; Instituto Rene Rachou, Fundação Oswaldo Cruz, Minas Gerais, Brazil; Climate Amplified Diseases and Epidemics (CLIMADE), Brazil, Americas; Unit of Medical Statistics and Molecular Epidemiology, Università Campus Bio-Medico di Roma, Rome, Italy; Department of Biomedical Sciences, University of Sassari, Sassari, Italy

**Keywords:** Macaca mulatta, Herpes B virus, Zoonosis, Surveillance, Public health

Dear Editor

Recently, the first documented case of B virus in humans in Hong Kong (https://tinyurl.com/2024-herpesbvirus-cases) has raised new concerns about a potential public health threat, given the notoriety of the pathogen and the worldwide presence of macaque monkeys, its natural hosts. The incident involved a 37-year-old man who experienced severe neurological symptoms after being scratched and bitten by wild macaques in a local park. B virus (also commonly referred to as herpes B, *herpesvirus simiae*, and herpesvirus) [1] is an extremely rare but potentially lethal infection in humans, with a historical mortality rate exceeding 70% in untreated cases [2]. The virus typically resides asymptomatically in macaque monkeys, including rhesus macaques, pig-tailed macaques and cynomolgus monkeys, which show no signs of disease despite being able to transmit the virus through saliva, urine or feces. The main mode of transmission to humans is through bites or scratches, which can cause severe neurological damage and death if not treated promptly and effectively. Human cases documented in the literature are summarized in Table 1. These cases involve a variety of macaque species, predominantly *Macaca mulatta*, which has been implicated in multiple incidents. This reflects the role of this species as the main natural host of the virus. Most human infections have occurred through bites or scratches, highlighting the danger of direct contact with these animals. However, cases resulting from apparently minor exposures, such as contact with saliva or handling of macaque tissues in a laboratory setting, have also been documented. Outcomes vary widely, from complete recoveries to deaths, depending on the speed of treatment and the nature of the exposure. The most severe cases tend to be those untreated or with delayed treatment. Incubation periods before symptom manifestation vary, but many cases show rapid disease expression within a few days of exposure. Symptoms range from mild skin complaints to severe neurological and respiratory complications.

**Table 1:** Human cases documented in the literature due to B virus.

Source	Year	Exposure	Monkey species	Incubation	Survival	Comments
*https://doi.org/10.1111/j.1600-0684.1987.tb00322.x*	1932	Bite, hand	*Macaca mulatta*	3 days	17 days	
*https://doi.org/10.1111/j.1600-0684.1987.tb00322.x*	1949	Saliva, hand	*Macaca mulatta*	Not reported	Few days	
*https://doi.org/10.1111/j.1600-0684.1987.tb00322.x*	1956	Scratch	Not reported	2 days	Recovered	Moderate neurological impairment
*https://doi.org/10.1111/j.1600-0684.1987.tb00322.x*	1957	Bite, hand	Not reported	35 days	30 days	
*https://doi.org/10.1111/j.1600-0684.1987.tb00322.x*	1957	Aerosol?	Probably *Macaca mulatta*	Unknown	7 days	A Doctor of Veterinary Medicine (DVM) who performed polio virus safey
*https://doi.org/10.1111/j.1600-0684.1987.tb00322.x*	1957	Cleaned skull	*Macaca mulatta*	14 days	2 days	A chemist with single known exposure to *Macaca mulatta*
*https://doi.org/10.1111/j.1600-0684.1987.tb00322.x*	1957	Bite, finger	*Macaca mulatta*	18 days	8 days	
*https://doi.org/10.1111/j.1600-0684.1987.tb00322.x*	1957	Bites, fingers	*Macaca mulatta*, *Macaca fascicularis*	13 days	Recovered	Mild neurological impairment
*https://doi.org/10.1111/j.1600-0684.1987.tb00322.x*	1957	Unknown	Not reported	Unknown	2 days	Monkey bites 4 months previously
*https://doi.org/10.1111/j.1600-0684.1987.tb00322.x*	1958	Cuts, hand	Not reported	2 days	15 days	Cut by monkey tissue culture bottle
*https://doi.org/10.1111/j.1600-0684.1987.tb00322.x*	1958	Needle puncture, hand	Not reported	5 days	7 days	
*https://doi.org/10.1111/j.1600-0684.1987.tb00322.x*	1958	Needle scratch, monkey bite, hand	Not reported	5 weeks	3 days	Two reported injuries
*https://doi.org/10.1111/j.1600-0684.1987.tb00322.x*	1960	Scratches, forearms	*Macaca mulatta*	Not reported	38 days	Throat blisters, early respiratory symptoms
*https://doi.org/10.1111/j.1600-0684.1987.tb00322.x*	1963	Aerosol?	*Macaca mulatta*, *Cercopithecus aethiops*	Unknown	Recovered	Severe neurological impairment; lived 3 yr, 4mo; isolate lethal to rabbits
*https://doi.org/10.1111/j.1600-0684.1987.tb00322.x*	1965	Bite	*Cercopithecus aethiops*	54 days	20 days	Biting monkey had increasing titer to B virus
*https://doi.org/10.1111/j.1600-0684.1987.tb00322.x*	1970	Recurrent?	*Macaca mulatta*; others?	Unknown	Recovered	Disease presented as zoster-like rash; severe neurological impairment; lived 12 y
*https://doi.org/10.1111/j.1600-0684.1987.tb00322.x*	1973	Bites?	Macaca mulatta, others	≥ 1 yr?	Recovered, 88-day illness	Minimal neurological problems, vision problems, impotence
*https://doi.org/10.1111/j.1600-0684.1987.tb00322.x*	1987	Bitten on left thumb	*Rhesus monkey*	18 days	Semi-comatose	Monkey suffering from severe bilateral conjunctivitis and diarrhea
*https://tinyurl.com/1987-herpesbvirus-cases*	1987	Penetrating wound on left forearm	Probably *Rhesus monkey*	Unknown	Died	Lesions initially diagnosed as herpes zoster or herpes simplex
*https://tinyurl.com/1987-herpesbvirus-cases*	1987	Clinically healthy monkey handling	Not reported	Not reported	Asymptomatic	Developed pruritic vesicles on finger, later healed
*https://tinyurl.com/1987-herpesbvirus-cases*	1987	Applied hydrocortisone cream	Not reported	Not reported	Asymptomatic	Developed pruritic dermatitis, tested positive for B-virus
*https://tinyurl.com/1989-herpesbvirus-cases*	1989	Monkey bites to hands, arms, and chest wall	*Macaca mulatta*, *Macaca fascicularis*	Unknown	Died	Symptoms included pain, numbness, dysesthesia, weakness, dizziness, difficulty swallowing, and respiratory arrest
*https://tinyurl.com/1989-herpesbvirus-cases*	1989	Monkey bite to right thumb	*Macaca mulatta*, *Macaca fascicularis*	Unknown	Clinical stable	Symptoms included fever, chills, headaches, myalgia, difficulty urinating, paresthesia, and dizziness
[5]	2021	Dissected dead monkeys	Not reported	1 month	Died	Symptoms include nausea, vomiting, fever, and neurological symptoms
*https://tinyurl.com/2024-herpesbvirus-cases*	2024	Contact with wild monkeys in Kam Shan Country Park	Rhesus Macaque *(Macaca mulatta),* Long-tailed Macaque *(Macaca fascicularis) and their hybrids*	Not reported	Critical condition	Diagnosed with B virus infection after being wounded by wild monkeys, admitted to Yan Chai Hospital with fever and decreased conscious level

The rarity of B virus infections in humans may lead to an underestimation of its potential threat. As human populations expand, more natural habitats are converted for agricultural or urban development. This encroachment forces wildlife into closer proximity with human communities, thereby increasing the chances of disease transmission. The clearing of forests for agriculture, logging, and urban expansion disrupts the natural habitats of many wildlife species, including macaques, which are natural carriers of the B virus. Deforestation not only reduces biodiversity but also increases the likelihood of wildlife entering human-populated areas, which can lead to more frequent interactions and potential transmission of zoonotic diseases. Activities such as eco-tourism, adventure travel, and the expansion of human settlements into previously undeveloped areas lead to greater direct and indirect contacts between humans and wildlife. Jones et al. [3] highlight that over 60% of emerging infectious diseases are zoonotic, originating primarily from wildlife. This statistic is a clear indicator of the growing challenge posed by these diseases as environmental degradation continues. The majority of these pathogens have the potential to cause significant health crises, yet the global preparedness and focus on these diseases often lag behind the immediate threats they could pose.

Preventive strategies against B virus are critical, especially for those who work in direct contact with primates or near their habitats. The Centers for Disease Control and Prevention (CDC) recommends specific protocols for handling macaques and their tissues in laboratories and zoos (https://www.cdc.gov/herpesbvirus/healthcare-providers.html). These guidelines include wearing protective clothing, immediate washing and disinfection of bites or scratches, and post-exposure prophylaxis with antivirals such as valacyclovir or acyclovir. Moreover, the development of educational programs to inform the public about the risks of direct contact with wild animals is essential. For example, the Hong Kong health authorities have already begun implementing measures to educate the public and prevent further incidents. These include posting warning signs in parks, regulating monkey populations, and conducting public health campaigns on the dangers of feeding and interacting with wild monkeys.

The introduction of decision-making tools for post-exposure antiviral prophylaxis (PEP), such as the one developed by Barkati et al. [4], shows a significant advance in the management of risk of B virus infection. This tool was developed after a comprehensive review of historical data on human infections, with the goal of simplifying the response to potential B virus exposures in a clinical setting. By classifying lesions into high-risk, moderate-risk, and low-risk categories based on several factors, including the type, depth, and location of exposure, clinicians can make informed decisions about the application of PEP. Ongoing training and reminders about proper animal handling protocols and the use of personal protective equipment are vital. The development and implementation of a structured decision tool for assessing and responding to B virus exposure risks represent a significant advancement in occupational health safety for primate workers. This approach not only enhances the immediate response to potential infections but also contributes to long-term preventive strategies by reducing unnecessary medical interventions and focusing resources on high-risk exposures.

As the interface between human and wildlife habitats becomes increasingly blurred, understanding and managing the environmental factors that contribute to the spread of zoonotic diseases such as B virus is more critical than ever. Mitigating the risks of B virus and other zoonotic diseases requires a multifaceted approach:

1. Strengthening surveillance: Monitoring disease patterns in both wildlife and human populations can contribute to early detection and response to potential outbreaks.
2. Educating the public: Informing people about the risks associated with contact with wildlife and the importance of maintaining a safe distance can reduce the number of direct interactions that lead to disease transmission.
3. Environmental management: Sustainable land use and forest conservation efforts are critical to preserving natural barriers between wildlife and human populations.
4. Health infrastructure: It is critical to strengthen health systems to respond more effectively to zoonotic diseases, including early detection, isolation, treatment, and widespread access to medical care.

Implementing rigorous management of these factors, alongside robust public health strategies, is paramount for substantially reducing associated risks and safeguarding global health.

## Funding

None.

## CRediT authorship contribution statement

**Francesco Branda:** Writing – review & editing, Writing – original draft, Investigation, Data curation, Conceptualization. **Alessandra Ciccozzi:** Writing – review & editing, Writing – original draft. **Marta Giovanetti:** Writing – review & editing, Writing – original draft. **Chiara Romano:** Writing – review & editing, Writing – original draft. **Massimo Ciccozzi:** Writing – review & editing, Writing – original draft, Validation, Supervision, Conceptualization. **Fabio Scarpa:** Writing – review & editing, Writing – original draft.

## Declaration of competing interest

The authors declare that they have no known competing financial interests or personal relationships that could have appeared to influence the work reported in this paper.
